# Allele-Specific Regulation of Matrix Metalloproteinase-3 Gene by Transcription Factor NFκB

**DOI:** 10.1371/journal.pone.0009902

**Published:** 2010-03-25

**Authors:** Veronika Souslova, Paul A. Townsend, Jelena Mann, Chris M. van der Loos, Anna Motterle, Fulvio D'Acquisto, Derek A. Mann, Shu Ye

**Affiliations:** 1 William Harvey Research Institute, Barts and The London School of Medicine and Dentistry, Queen Mary University of London, London, United Kingdom; 2 Human Genetics Division, MP808, School of Medicine, University of Southampton, Southampton, United Kingdom; 3 Institute of Cellular Medicine, Newcastle University, Newcastle, United Kingdom; 4 Department of Pathology, Academic Medical Centre, Amsterdam, The Netherlands; Ohio State University Medical Center, United States of America

## Abstract

**Background:**

Matrix metalloproteinase-3 (MMP3) is implicated in the pathogenesis and progression of atherosclerotic lesions. Previous studies suggested that *MMP3* expression is influenced by a polymorphism (known as the 5A/6A polymorphism) in the promoter of the *MMP3* gene and that this polymorphism is located within a *cis*-element that interacts with the transcription factor NFκB. In the present study, we sought to investigate whether MMP3 and NFκB were co-localized in atherosclerotic lesions and whether NFκB had differential effects on the two alleles of the *MMP3* 5A/6A polymorphism.

**Methodology/Principal Findings:**

Immunohistochemical examination showed that MMP3 and both the NFκB p50 and p65 subunits were expressed abundantly in macrophages in atherosclerotic lesions and that MMP3 expression was co-localized with p50 and p65. Chromatin immunoprecipitation experiments showed interaction of p50 and p65 with the *MMP3* promoter in macrophages, with greater binding to the 5A allele than to the 6A allele. Reporter gene assays in transiently transfected macrophages showed that the 5A allele had greater transcriptional activity than the 6A allele, and that this allele-specific effect was augmented when the cells were treated with the NFκB activator lipopolysaccharides or co-transfected with p50 and/or p65 expressing plasmids, but was reduced when the cells were treated with the NFκB inhibitor 6-Amino-4-(4-phenoxyphenylethylamino)-quinazoline or transfected with a dominant negative mutant of IkB kinase-β.

**Conclusion:**

These results corroborate an effect of the 5A/6A polymorphism on *MMP3* transcription and indicate that NFκB has differential effects on the 5A and 6A alleles.

## Introduction

Atherosclerotic plaque rupture is the most common cause of acute clinical ischemic events associated with coronary artery disease [1]. A typical atherosclerotic plaque contains a lipid core covered by a fibrous cap, and plaque rupture is mostly the result of fibrous cap fissuring [1], [2]. There is evidence indicating that increased expression of matrix metalloproteinase-3 (MMP3) in the atherosclerotic plaque promotes plaque rupture [3]–[5]. MMP3 is abundantly expressed by macrophages in atherosclerotic plaques, particularly in the lateral regions of the fibrous cap where fissuring is most likely to occur [3], [4]. This protease is capable of degrading several major structural matrix proteins present in atherosclerotic plaques [6], [7]. A study of a mouse model of atherosclerosis has shown that knocking out MMP3 results in increased amounts of matrix proteins in atherosclerotic lesions, a feature of more stable plaques [5].

The transcription of the *MMP3* gene is tightly regulated [8]. We and others have shown that its transcription is influenced by a polymorphism in the promoter of the gene [9], [10]. The polymorphism (dbSNP ID rs3025058), known as the 5A/6A polymorphism, is due to a single base deletion (or insertion) in a run of thymidines, resulting in one allele (the 5A allele) having 5 thymidines and the other allele (the 6A allele) having 6 thymidines, at nucleotide positions from −1608 to −1612 (or −1613) relative to the transcription start site. Previously studies by our and other groups have demonstrated that the 5A allele has greater promoter activity than the 6A allele [9], [10]. In agreement, it has also been shown that MMP3 mRNA and protein levels in arterial tissues are higher in individuals who are homozygous for the 5A allele than in those who are homozygous for the 6A allele, with intermediate levels in heterozygous individuals [11]. Genetic epidemiological studies have shown an association between the 5A allele and increased risk of myocardial infarction and several other cardiovascular disorders [12]–[15].

*MMP3* transcription is modulated by the transcription factor NFκB [16]–[19] and there is evidence indicating that the DNA sequence encompassing the 5A/6A polymorphic site can interact with NFκB [20]. In the present study, we sought to investigate whether MMP3 and NFκB were co-expressed in atherosclerotic lesions and to characterize the regulatory effect of NFκB on the *MMP3* gene 5A and 6A alleles.

## Results

**Figure 1:**
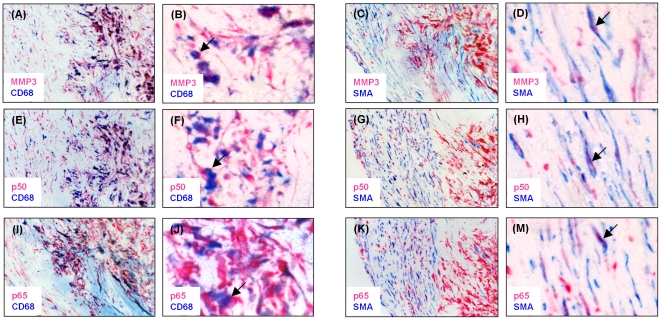
Macrophages and smooth muscle cells in atherosclerotic plaques express MMP3 and NFκB. Sections (4 µm) of paraffin embedded tissue blocks of atherosclerotic plaques were subjected to double immunostaining of MMP3 and the macrophage marker CD68 (A and B), MMP3 and the smooth muscle cell marker SMA (C and D), p50 and CD68 (E and F), p50 and SMA (G and H), p65 and CD68 (I and J), p65 and SMA (K and M), using rabbit polyclonal NFκB p50 and MMP3 antibodies (Abcam), a rabbit monoclonal NFκB p65 antibody (Abcam), and mouse monoclonal CD68 and smooth muscle actin (SMA) antibodies (DAKO), respectively, and goat anti-rabbit or anti-mouse secondary antibodies (Thermo/LabVision). The double immunostaining was visualized with Liquid Permanent Red chromogen (Dako) and Vector Blue chromogen (Vector Labs). Pink colour indicates expression of MMP3 (A, B, C and D), p50 (E, F, G and H) or p65 (I, J, K and M). Blue colour indicates expression of CD68 (A, B, E, F, I and J) or SMA (C, D, G, H, K and M). Arrow indicates cell co-expressing MMP3 with CD68 (B) or SMA (D), p50 with CD68 (F) or SMA (H), and p65 with CD68 (J) or SMA (M). 200X magnification in A, C, E, G, I and K; 400X magnification in B, D, F, H, J and M.

**Figure 2:**
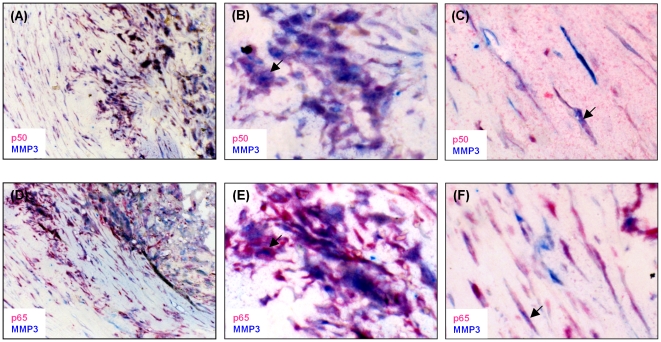
MMP3 and NFκB are co-localized in atherosclerotic plaques. Sections (4 µm) of paraffin embedded tissue blocks of atherosclerotic plaques were subjected to double immunostaining of MMP3 and p50 (A, B, and C) or MMP3 and p65 (D, E and F), using rabbit polyclonal NFκB p50 and MMP3 antibodies (Abcam) and a rabbit monoclonal NFκB p65 antibody (Abcam), respectively, and goat anti-rabbit secondary antibodies (Thermo/LabVision). The double immunostaining was visualized with Liquid Permanent Red chromogen (Dako) and Vector Blue chromogen (Vector Labs). Pink colour indicates expression of p50 (A, B and C) or p65 (D, E and F). Blue colour indicates expression of MMP3 (A, B, C, D, E and F. Arrow indicates cell co-expressing MMP3 with p50 (B and C) or with p65 (E and F). 200× magnification in A and D; 400× magnification in B, C, E and F.

We carried out an immunohistochemical analysis to determine whether MMP3 and NFκB were co-expressed in atherosclerotic lesions. The analysis showed that MMP3 and both the NFκB p50 and p65 subunits were expressed abundantly in macrophages in atherosclerotic lesions and were also present in smooth muscle cells in these tissues (Figure 1). Importantly, we found that MMP3 expression was co-localized with p50 and p65 in these lesions, although this was not seen in every cell (Figure 2).

**Figure 3:**
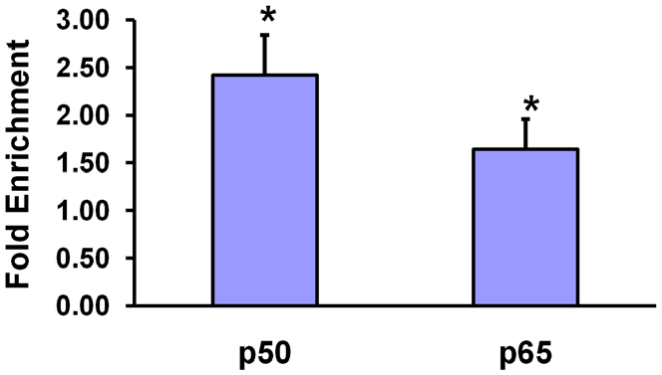
Interaction of p50 and p65 with MMP3 gene promoter. Chromatin proteins and DNA in THP1 cells were cross-linked in 1% formaldehyde for 10 minutes. Cells were then lysed, and the lysate sonicated to reduce DNA length to 200–1000 bp. The sonicated chromatin was subjected to immunoprecipitation using a p50 or p65 antibody, with rabbit IgG as a control, followed by PCR amplification of 406 bp and 407 bp DNA fragments surrounding the *MMP3* gene 5A/6A site and then agarose gel electrophoresis of the PCR products. Intensity of bands on the agarose gels were quantified with the computer software ImageJ. The fold enrichment of DNA precipitated with p50 antibody or p65 antibody, relative to the IgG control, was calculated by comparing the intensity of the band of the amplicon from the p50 or p65 antibody precipitate to the intensity of the band of the amplicon from the IgG control. The graph shows fold enrichments of DNA precipitated with p50 antibody or p65 antibody, relative to the IgG control. Data shown are mean (± standard error of mean) from four independent experiments. * denotes p<0.05 comparing DNA precipitated with p50 antibody or p65 antibody to the IgG control.

We then performed chromatin immunoprecipitation experiments in THP1 monocytes to study whether NFκB could interact with the *MMP3* gene promoter. The experiments showed binding of p50 and p65 to the *MMP3* promoter in these cells (Figure 3).

**Figure 4:**
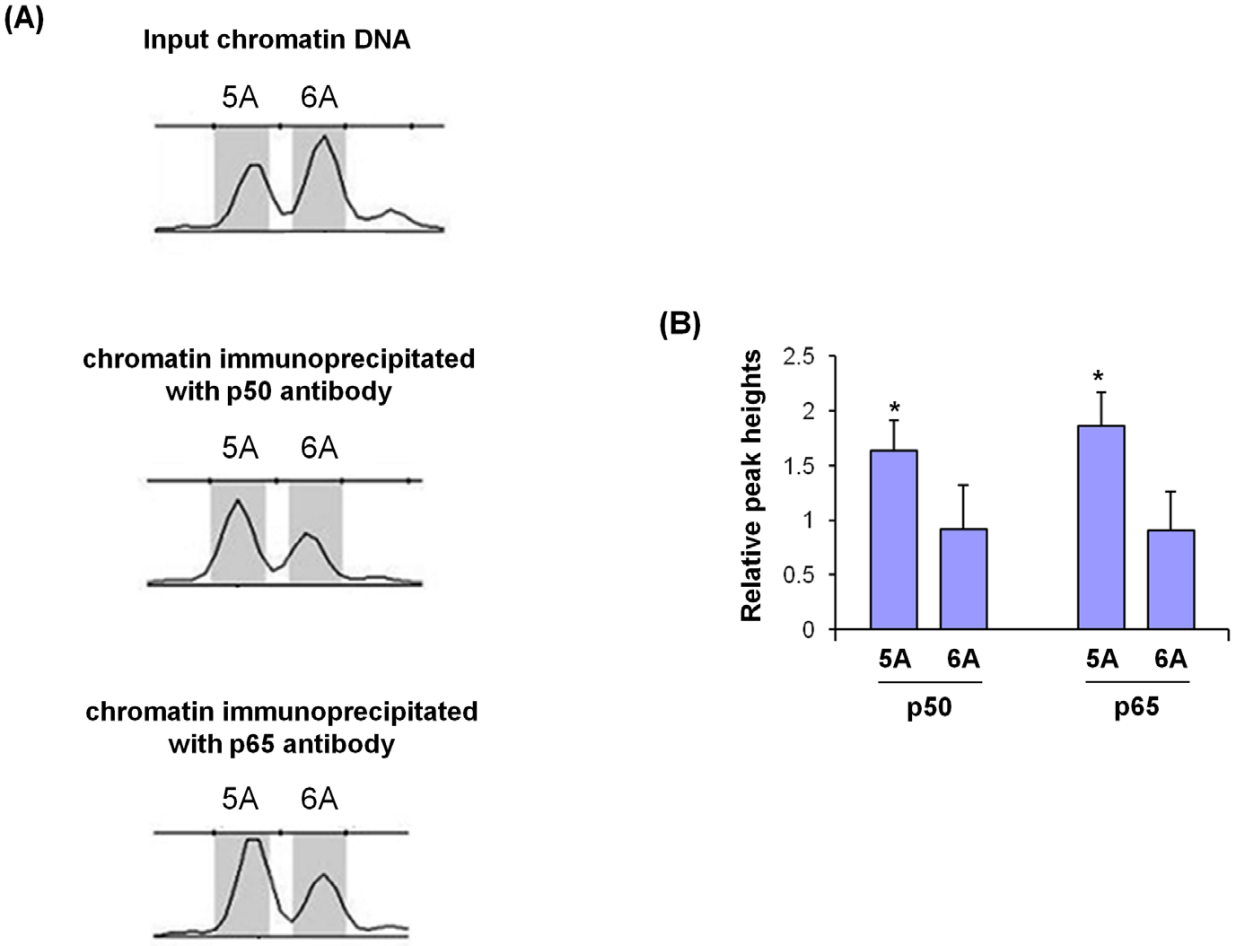
The MMP3 gene 5A allele more readily interacts with p50 and p65, than the 6A allele. Chromatin proteins and DNA in THP1 cells were cross-linked in1% formaldehyde for 10 minutes. Cells were then lysed, and the lysate sonicated to reduce DNA length to 200–1000 bp. The sonicated chromatin was subjected to immunoprecipitation using a p50 or p65 antibody, or incubated with a rabbit IgG, followed by PCR amplification of 179 bp and 180 bp DNA fragments surrounding the *MMP3* gene 5A/6A site using fluorescence labelled primers. The PCR amplicons were analyzed using an ABI 3730xl analyzer and Genemapper software. Three independent experiments were performed. (A). Representative output from the Genemapper programme. The panels from top to bottom represent input chromatin DNA, chromatin immunoprecipitated with the p50 antibody, and chromatin immunoprecipitated with the p65 antibody. (B). Chart shows mean (± standard error of mean) of the relative peak heights of the 5A and 6A alleles in the p50 and p65 antibody precipitates, standardized against the relative peak heights of the 5A and 6A alleles in the input DNA, in three independent experiments. * denotes p<0.05 comparing 5A versus 6A alleles.

We then investigated whether the *MMP3* 5A/6A polymorphism had an effect on NFκB binding. Using the THP1 cell line (these cells are heterozygous for the *MMP3* 5A/6A polymorphism), we compared the relative amounts of chromatins of the 5A and 6A alleles respectively, precipitated using anti-NFκB antibodies. We found that both an anti-p50 antibody and an anti-p65 antibody precipitated greater amount of 5A allele chromatin than 6A allele chromatin (Figure 4), suggesting that NFκB interacted more readily with the 5A allele than the 6A allele.

**Figure 5:**
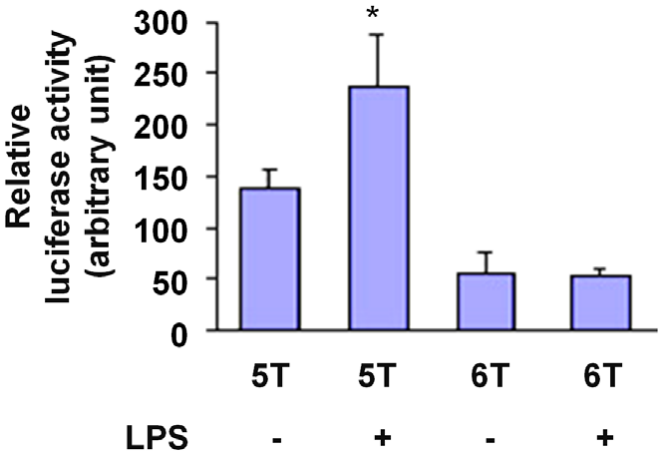
Lipopolysaccharide increases 5A allele activity. RAW264 cells were transfected with an *MMP3* 5A-firefly luciferase reporter plasmid or an *MMP3* 6A-firefly luciferase reporter plasmid, and the pRL-TK plasmid (expressing *renilla* luciferase) to serve as a reference for transfection efficiency. Transfected cells were cultured for 24 hours in the absence or presence of lipopolysaccharides. Luciferase reporter assay was performed using a dual-luciferase reporter assay system. Data shown are mean (± standard error of mean) of the ratio of firefly luciferase activity over *renilla* luciferase activity from four independent experiments. Asterisk indicates p<0.05 comparing untreated 5A and LPS treated 5A.

To further characterize the effect of NFκB on *MMP3* 5A and 6A alleles, we performed transfection and luciferase reporter assays in which macrophages were transfected with plasmids containing a *MMP3* gene promoter sequence corresponding to either the 5A or 6A allele and a firefly luciferase reporter gene, and treated the transfected cells with lipopolysaccharide (LPS), an endotoxin stimulus of NFκB activation. These experiments demonstrated that without LPS stimulation, luciferase activity in cells transfected with the 5A allele construct was 2.4 fold greater than that in cells transfected with the 6A allele construct (p<0.01), confirming that the 5A allele has higher promoter activity than the 6A allele (Figure 5). More importantly, the experiments showed that in cells transfected with the 5A allele construct, luciferase activity was increased by LPS treatment, whereas in cells transfected with the 6A allele construct, LPS treatment did not increase luciferase activity (Figure 5), which is consistent with the notion that NFκB acts more effectively on the 5A allele than the 6A allele.

**Figure 6:**
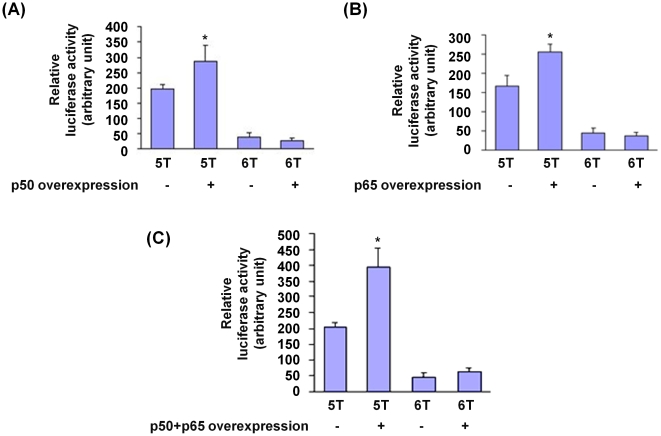
NFκB has a greater upregulatory effect on the 5A allele than the 6A allele. RAW264 cells were transfected with an *MMP3* 5A-firefly luciferase reporter plasmid or an *MMP3* 6A-firefly luciferase reporter plasmid, and the pRL-TK plasmid (expressing *renilla* luciferase) to serve as a reference for transfection efficiency, together with a plasmid expressing p50 and/or a plasmid expressing p65. Luciferase reporter assay was performed using a dual-luciferase reporter assay system. Data shown are mean (± standard error of mean) of the ratio of firefly luciferase activity over *renilla* luciferase activity from four independent experiments. In panel (A), * indicates p = 0.07 comparing 5A and 5A plus p50. In panel (B), ** indicates p<0.01 comparing 5A and 5A plus p65. In panel (C), * denotes p<0.05 comparing 5A with 5A plus p50 and p65.

To verify that NFκB has differential effects on the 5A and 6A alleles, we co-transfected macrophages (RAW264 cells) with the 5A or 6A construct together with p50 and/or p65 expressing plasmids. The experiment showed that over-expressing p50 or p65 resulted in an increase in luciferase activity in cells transfected with the 5A construct and this upregulatory effect was further increased when both the p50 and p65 subunits were overexpressed (Figure 6). In contrast, in cells transfected with the 6A construct, there was only a small increase in luciferase activity only when both the p50 and p65 subunits were overexpressed (Figure 6).

**Figure 7:**
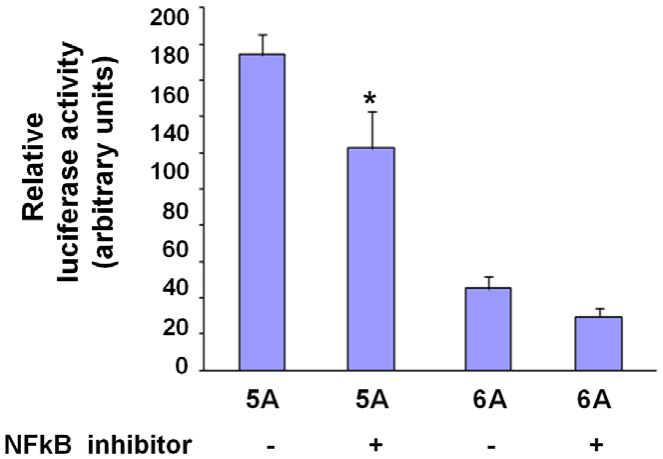
NFκB inhibitor has a suppressive effect. RAW264 cells were transfected with an *MMP3* 5A-firefly luciferase reporter plasmid or an *MMP3* 6A-firefly luciferase reporter plasmid, and the pRL-TK plasmid (expressing *renilla* luciferase) to serve as a reference for transfection efficiency. Transfected cells were cultured for 24 hours in the absence or presence of the NFκB inhibitor 6-Amino-4-(4-phenoxyphenylethylamino)-quinazoline (10 µM concentration). Luciferase reporter assay was performed using a dual-luciferase reporter assay system. Data shown are mean (± standard error of mean) of the ratio of firefly luciferase activity over *renilla* luciferase activity from three independent experiments. * indicates p<0.05 comparing 5A with 5A plus NFκB inhibitor.

To further verify the effect of NFκB, we examined whether the NFκB inhibitor 6-Amino-4-(4-phenoxyphenylethylamino)-quinazoline had an effect on luciferase activity in cells transfected with the 5A or 6A construct. The experiment showed that the NFκB inhibitor reduced luciferase activity, most notably in cells transfected with the 5A allele construct (Figure 7).

**Figure 8:**
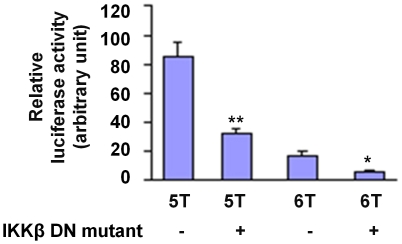
Expressing a dominant negative mutant of IKKβ has a suppressive effect. RAW264 cells were transfected with an *MMP3* 5A-firefly luciferase reporter plasmid or an *MMP3* 6A-firefly luciferase reporter plasmid, and the pRL-TK plasmid (expressing *renilla* luciferase) to serve as a reference for transfection efficiency, together with a plasmid expressing a dominant negative mutant of IKKβ. Luciferase reporter assay was performed using a dual-luciferase reporter assay system. Data shown are mean (± standard error of mean) of the ratio of firefly luciferase activity over *renilla* luciferase activity from three independent experiments. ** indicates p<0.01 comparing 5A with 5A plus IKKbeta dominate negative mutant; and * indicates p<0.05 comparing 6A with 6A plus IKKbeta dominate negative mutant.

Since NFκB is activated through the action of IkB kinases (IKK), we investigated whether suppression of IKK activity would have differential effects on the 5A and 6A alleles. The experiment showed that expression of a dominant negative mutant of IKKβ resulted in a significant reduction in luciferase activity, especially in cells transfected with the 5A allele construct which showed a 60% reduction (p<0.01, Figure 8).

## Discussion

Our study demonstrates that MMP3 and NFκB are colocalized in macrophages and smooth muscle cells in atherosclerotic plaques. With the use of the chromatin immunoprecipitation technique, we showed that NFκB can interact with the *MMP3* gene promoter and that this transcription factor binds more readily to the 5A allele than the 6A allele. In line with this finding, reporter assays in transiently transfected macrophages showed that the 5A allele has greater transcriptional activity than the 6A allele, and that this allele-specific effect was augmented in cells treated with the NFκB activator LPS or co-transfected with p50 and/or p65 expressing plasmids, but was reduced when the cells were treated with the NFκB inhibitor 6-Amino-4-(4-phenoxyphenylethylamino)-quinazoline or transfected with a dominant negative mutant of IkB kinase-β. Taken together, these results indicate that the 5A/6A polymorphism influences *MMP3* transcription due to differential effects of NFκB on the 5A and 6A alleles.

MMP3 has proteolytic activity on types III, IV and V collagen, proteoglycans, laminin, fibronectin and elastin [6], [7], which are the principal components of the extracellular matrix of the arterial wall and provide the strength of the atherosclerotic plaque to withstand the mechanical stress imposed on it by the blood circulation [21]. In addition, MMP3 is an activator of several other MMPs including MMP-1, -7, -8, -9 and -13 [22]–[24] which are also important players in atherogenesis and plaque rupture [4], [25]–[28]. There is evidence indicating that increased MMP3 expression in atherosclerotic plaque renders the plaque prone to rupture [3]–[5]. Plaque rupture is the most common cause of acute clinical ischemic events such as myocardial infarction and stroke [1]. Thus, an insight into the molecular mechanisms that govern the regulation of MMP3 expression will enhance our understanding of the pathogenesis of atherosclerosis and plaque rupture.

It is now widely accepted that atherosclerosis is a chronic inflammatory disease of the vessel wall [29]. NFκB is a key regulator of inflammation and can mediate the induction of more than 160 genes, many of which have a documented role in atherosclerosis [30]. Previous studies have shown that NFκB plays an important role in the regulation of MMP3 expression [16]–[19]. However, the element(s) in the MMP3 gene that mediate(s) the regulatory effect of NFκB had not yet been identified. Our present study indicates that the sequence encompassing the 5A/6A site is likely to be an NFκB responsive site.

The 5A/6A polymorphism has been associated with risk of myocardial infarction [12], [13], [31]. In addition, it has been associated with several other cardiovascular diseases including cardiac mortality in patients with heart failure [32], coronary aneurysm [33], restenosis after coronary angioplasty [34], [35], carotid atherosclerosis and stroke [36]–[38]. Studies have shown that MMP3 mRNA and protein levels in *ex vivo* tissues including arteries are highest in 5A homozygotes, intermediate in heterozygotes and lowest in 6A homozygotes [11], [39], and that the 5A allele has greater promoter activity and *MMP3* transcription than the 6A allele [9], [10], although the molecular mechanism underlying the effect of this polymorphism on *MMP3* gene promoter activity and transcription is unclear. The results of the present study corroborate the regulatory effect of the 5A/6A polymorphism and indicate that this effect is likely to derive from differential effects of NFκB on the 5A and 6A alleles.

In conclusion, the findings of this study are highly relevant to the understanding of the transcriptional regulation of *MMP3*, increased expression of which has been suggested to play an important role in atherosclerotic plaque rupture [3]–[5]. In addition, they provide an insight into the functional effect of the 5A/6A polymorphism which has been associated with a number of cardiovascular traits including risk of myocardial infarction [14].

## Materials and Methods

### Chemicals

Lipopolysaccharide was obtained from Sigma. 6-Amino-4-(4-phenoxyphenylethylamino)- quinazoline was from Merck Chemicals Ltd. Antibodies for p50 (ab7971), p65 (ab32536 and ab7970) and MMP3 (ab53015) were from Abcam. ChIP assay kit was from Upstate Biotechnology.

### Immunohistochemistry

Immunohistochemical analyses were performed on 4 µm sections of formalin-fixed and paraffin-embedded tissue blocks of atherosclerotic plaques removed from patients undergoing carotid endarterectomy. The study was approved by the South and West Local Research Ethics Committee, and all participants provided written informed consent. Tissue sections were pretreated with Tris-EDTA pH 9.0. Immunostaining was carried out as described previously [40]. We used rabbit polyclonal NFκB p50 (ab7971, Abcam) and MMP3 antibodies (ab53015, Abcam), a rabbit monoclonal NFκB p65 antibody (ab32536, Abcam), and mouse monoclonal CD68 (clone PG-M1) and smooth muscle alpha-actin (SMA, clone 1A4) antibodies (Dako, Glostrup, Denmark), respectively. Secondary antibodies were goat anti-mouse IgG or anti-rabbit IgG/alkaline phosphatase (AP) polymers (Thermo/LabVision, Fremont, CA). The sequential AP double immunostaining was performed as previously described [41] with Liquid Permanent Red (Dako) and Vector Blue used as chromogens (Vector Labs, Burlingame, CA).

### Cell culture

THP1 and RAW264 cells were purchased from the American Type Culture Collection (ATCC) and the European Collection of Cell Cultures (ECACC), respectively. THP1 cells were maintained in RPMI-1640 medium (ATCC) supplemented with 10% foetal bovine serum (Invitrogen), 2 mM L-glutamine (Invitrogen), 50 U/ml penicillin and 50 µg/ml streptomycin (Invitrogen). RAW264 cells were maintained in DMEM medium (Sigma) supplemented with 10% foetal bovine serum (Invitrogen), 2 mM L-glutamine (Invitrogen), 50 U/ml penicillin and 50 µg/ml streptomycin (Invitrogen). The cells were incubated at 37°C in a cell incubator supplied with 5% CO_2_.

### Chromatin immunoprecipitation

Chromatin immunoprecipitation was carried out by using a chromatin immunoprecipitation kit from Upstate Biotechnology according to the manufacturer manual. Briefly, THP1 cells were grown to density at 0.8–1×10^6^ cells per ml of culture medium. Proteins were cross-linked to DNA by adding 37% of formaldehyde directly to the medium to a final concentration of 1%, and after 10 minutes of incubation at room temperature, unreacted formaldehyde was quenched with 0.125 M glycine for 5 minutes. Cells were pelleted, washed 2–3 times in ice-cold phosphate buffered saline and lysed in lysis buffer with protease inhibitors for 10 minutes on ice. The lysate was sonicated to reduce DNA length to 200–1000 bp and then spun at 10,000 g at 4°C for 10 minutes to remove insoluble materials. Immunoprecipitation of chromatin was performed by incubation with 2 µg anti-p50 antibody (ab7971, Abcam) or 2 µg anti-p65 antibody (ab7970, Abcam) at 4°C for 18 hours. Incubation with 2 µg rabbit IgG was used as a control. Following the immunoprecipitation, PCR was carried out to amplify the DNA sequence surrounding the *MMP3* gene 5A/6A site, followed by agarose gel electrophoresis of the PCR products. Intensity of bands on the agarose gels were quantified with the computer software ImageJ. The fold enrichment of DNA precipitated with p50 antibody or p65 antibody, relative to the IgG control, was calculated by comparing the intensity of the band of the amplicon from the p50 or p65 antibody precipitate to the intensity of the band of the amplicon from the IgG control. Four independent experiments were carried out.

### Analysis of chromatin immunoprecipitation products

To detect chromatin immunoprecipitates, purified DNA was analyzed by PCR using specific *MMP3* promoter primers covering the 5A/6A polymorphic site: 5′-AATTCACATCACTGCCACCA-3′ and 5′-CTCTGTGGCAATAAGATCCC-3′, and PCR products were analysed on 2% agarose gels with GelRed staining. Intensity of bands on the agarose gels were quantified with the computer software ImageJ.

To compare the relative amounts of the 5A and 6A alleles in the chromatin immunoprecipitates, purified DNA obtained from chromatin immunoprecipitation was subject to PCR with a 6-FAM-labelled forward primer (5′-AATTCACATCACTGCCACCA-3′) and an unlabelled reverse primer (5′-GATTACAGACATGGGTCACG-3′) flanking the 5A/6A polymorphic site, with expected amplicons of 179 bp from the 5A allele and 180 bp from the 6A allele. The PCR reactions were carried out using Phire Hot Start DNA polymerase to generate blunt ended PCR products. The fluorescence labelled PCR fragments were analyzed using an ABI 3730xl analyzer and Genemapper software. Three independent experiments were performed. The relative peak heights of the 5A and 6A alleles in the p50 and p65 antibody precipitates were standardized against the relative peak heights of the 5A and 6A alleles in the input DNA and compared by a t-test.

### Plasmids

The MMP3 5A and 6A pGL3 reporter constructs were produced by cloning three copies of the 5A or 6A polymorphic site of the human MMP3 gene: 5′-ACAAGACATGGTTTTT(T)CCCCCCATCAAAGA-3′ into the pGL3 promoter vector (Promega), upstream of the SV40 promoter and the luciferase gene. NFκB p50, NFκB p65 and p300 expressing plasmids have been described [42]–[44]. The IKKβ dominate negative plasmid has been described previously [45].

### Transfection and luciferase reporter assay

The day before transfection, RAW264 cells were plated into 24-well tissue culture dishes at a density reaching 70–80% confluence by the following day. Cells were transfected with the *MMP3* 5A-luciferase reporter plasmid or the *MMP3* 6A-luciferase reporter plasmid, and the pRL-TK plasmid (Promega) to serve as a reference for transfection efficiency. Transfected cells were cultured for 24 hours in the absence or presence of LPS (100 ng/ml) or 6-Amino-4-(4-phenoxyphenylethylamino)-quinazoline (10 µM). In some experiments, the cells were also transfected with a p50 expression plasmid and/or a p65 expression plasmid, or with a pcDNA3 plasmid to serve as a control, or with a plasmid expressing a dominant negative mutant of IKKβ. Transfections were performed in duplicates with FuGENE 6 transfection reagent (Roche) using a protocol provided by the supplier. Luciferase reporter assay was performed using a dual-luciferase reporter assay system (Promega). Transfections and cell treatments were performed in duplicate and individual experiments were repeated at least three times.

### Statistical analysis

In the chromatin immunoprecipitation assays, the PCR band itensities of DNA precipitated with p50 antibody or p65 antibody were compared with the IgG control by Mann-Whitney test. The relative peak heights of the 5A and 6A alleles in the p50 and p65 antibody precipitates in the Genemaper programme outputs were standardized against the relative peak heights of the 5A and 6A alleles in the input DNA and compared by a t-test. In the luciferases reporter assays, t-tests were performed to compare differences between the 5A and 6A alleles and between different the treated and untreated. All data are represented as the mean ± standard error of mean (SEM).
